# The Prevalence of Symptomatology and Risk Factors in Mental Health in Mexico: The 2016–17 ENCODAT Cohort

**DOI:** 10.3390/ijerph20043109

**Published:** 2023-02-10

**Authors:** María Elena Medina-Mora, Alma Delia Genis-Mendoza, Jorge Ameth Villatoro Velázquez, Marycarmen Bustos-Gamiño, Clara Fleiz Bautista, Beatriz Camarena, José Jaime Martínez-Magaña, Humberto Nicolini

**Affiliations:** 1 Global Studies Seminar, School of Medicine, National Autonomous University of Mexico (UNAM), Mexico City 04510, Mexico; 2 Director, School of Psychology, National Autonomous University of Mexico (UNAM), Mexico City 04510, Mexico; 3 Centro de Investigación en Salud Mental Global INPRFM UNAM, Mexico City 04510, Mexico; 4 Psychiatric and Neurodegenerative Disease Genomics Laboratory, National Institute of Genomic Medicine (INMEGEN), Mexico City 14610, Mexico; 5 Juan N. Navarro Children’s Psychiatric Hospital, Psychiatric Care Services, Mexico City 14080, Mexico; 6 Data Analysis and Survey Unit, Ramón de la Fuente Muñiz National Institute of Psychiatry (INPRFM), Mexico City 14370, Mexico; 7 Department of Pharmacogenetics, Ramón de la Fuente Muñiz National Institute of Psychiatry (INPRFM), Mexico City 14370, Mexico

**Keywords:** prevalence, symptomatology, Mexican, OCD

## Abstract

There is little recent information about the prevalence of symptomatology of mental health disorders in representative population samples in Mexico. To determine the prevalence of mental health symptoms in Mexico and its comorbidity with tobacco, alcohol, and drug use disorder (SUD), we used the 2016–17 National Survey of Drug, Alcohol, and Tobacco Use (Encuesta Nacional de Consumo de Drogas, Alcohol y Tabaco, ENCODAT 2016–2017). The data were collected from households using a cross-sectional, stratified, multistage design, with a confidence level of 90% and a response rate of 73.6%. The final sample included 56,877 completed interviews of individuals aged 12–65, with a subsample of 13,130 who answered the section on mental health. Symptoms of mania and hypomania (7.9%), depression (6.4%), and post-traumatic stress (5.7%) were the three main problems reported. Of this subsample, 56.7% reported using a legal or illegal drug without SUD, 5.4% reported SUD at one time on alcohol, 0.8% on tobacco, and 1.3% on medical or illegal drugs, 15.9% reported symptoms related to mental health, and 2.9% comorbidity. The prevalence found is consistent with those reported in previous studies, except for an increase in post-traumatic stress, which is consistent with the country’s increase in trauma.

## 1. Introduction

Mental health disorders are among the health problems associated with the greatest levels of disability [1]. Mood disorders, anxiety disorders (including specific phobias), and alcohol use disorders are the most prevalent worldwide [1]. Among the risk factors that increase the probability of experiencing these disorders are low educational level, violence, low socioeconomic level, polygenic load, problems at work, and interpersonal problems. There may also be increased risk from having presented one or more mental health difficulties or disorders in early childhood or adolescence [2,3]. The National Health Survey in Mexico (Encuesta Nacional de Salud, ENSANUT, 2018) includes questions about depression symptomatology [4], and the national addiction surveys include questions about suicide attempts and emotional distress [5].

Surveys from various countries, including Mexico, show a lifetime prevalence of mental disorders according to DSM-IV criteria, with range of 47.4% and 12.0% in the U.S. and Nigeria, respectively. The extremes for the prevalence of anxiety and depression disorders are in the U.S. (31% and 21%, respectively) and China (4.8% and 3.6%, respectively). Disorders of impulse control are highest in the U.S. (25%) and the lowest in Nigeria (0.3%) [1]. The prevalence of substance use disorders is highest in Ukraine (15%) and lowest in Italy (1.3%) [1]. Glantz et al. (2020) [6] found a range in the prevalence of alcohol use disorder (AUD) from 0.2% in Iraq to 6% in the U.S. and from 0.5% in Iraq to 18.7 % in Australia for alcohol abuse.

Auerbach et al. (2018) evaluated the prevalence of mental illness in 19 universities in eight countries, including Mexico, and estimated that 35% of those surveyed suffered from at least one disorder such as major depression, mania/hypomania, generalized anxiety disorder, panic disorder, alcohol use disorder, or substance use disorder [7]. In Mexico, the most recent national survey of psychiatric epidemiology was in 2002, when the prevalence of psychiatric disorders was estimated as 30.4% in men and 27.1% in women. That survey reported that 6.7% of men and 11.2% of women had presented an affective disorder (depression, mania, hypomania, or dysthymia) at least once in their lives, 9.5% of men and 18.5% of women an anxiety disorder (panic, agoraphobia, social phobia, specific phobia, generalized anxiety, or post-traumatic stress), 17.6% of men and 2.0% of women a substance use disorder, 11.5% of men and 1.0% of women AUD, 0.7% of men and 0.2% of women drug use disorder (DUD), and 2.9% of men and 0.9% of women nicotine use disorder (NUD) [2].

Benjet et al. (2009) found that the prevalence is lower in adolescents, but that the more common disorders were specific phobias and social phobia. In women, the more common disorders were phobias, major depression, oppositional defiant disorder, agoraphobia without panic disorder, and separation anxiety; in men, they were oppositional defiant disorder, alcohol abuse, and conduct disorder. In general, women presented a greater number of disorders and a greater prevalence of each [3]. It is important to note that, to date, neither the frequency of obsessive-compulsive disorder nor that of post-traumatic stress disorder has been reported on the populational level.

Despite various efforts, there has been no more recent study in Mexico of the state of the mental health of the population, but updated data are important for the improvement of care and treatment. The objective of this study is, thus, to determine the prevalence of mental illness symptomatology in Mexico and its comorbidity with the use of tobacco, alcohol, and drugs, obtained through the 2016–17 National Survey of Drugs, Alcohol, and Tobacco Use (Encuesta Nacional de Consumo de Drogas, Alcohol y Tabaco 2016–17, ENCODAT 2016–17).

## 2. Materials and Methods

The study was carried out on a subsample of the National Survey of Drug, Alcohol, and Tobacco Use, administered in 2016 to participants aged 12–65 in urban and rural Mexican households [5]. The objective of the survey was to evaluate the patterns of use of different psychoactive substances and mental health problems in Mexico. The sample had a cross-sectional, stratified, probabilistic, and multistage design, with a confidence level of 90% and a response rate of 73.6%. The sample universe was made up of primary sampling units (PSUs) that were the sum of basic statistical geographic areas (BSGAs; Áreas Geográficas Estadísticas Básicas), stratified by state and urban–rural community. An adult aged 18–65 was chosen at random in each household, and in those with at least one household member under 18, an adolescent aged 12–17 was chosen at random. The persons chosen were administered individual questionnaires. The final sample included 56,877 completed interviews: 27,463 men and 29,414 women; 9563 adolescents and 47,314 adults.

The second administration of the survey included a subsample of 13,130 respondents who agreed to give a saliva sample for DNA analysis, to be used in analyzing the relationship of mental health, addiction, and genetics. This sample was weighted according to selection probability to be representative on the national level. Mental health symptomatology for these individuals was evaluated using the Diagnostic Interview for Psychosis and Affective Disorders (DI-PAD) using the operational criteria for psychotic disorders (OPCRIT v. 4.0), administered by interviewers trained in its use. This scale has shown a high level of consensus among interviewers (kappa = 0.8), with the best consensus for estimations of the presence of disorders during the lifetime of the interviewee [8]. It includes the evaluation of symptomatology of psychosis, depression, anxiety, obsession/compulsion, mania/hypomania, and post-traumatic stress [9]. The analysis also employed data taken from the first stage of the survey related to suicide attempts and pathological gambling [10,11]. As not all of the indicators of every aspect of mental health were included, the data were considered to represent symptoms of different illnesses, not definitive diagnoses.

### 2.1. Measurements

#### 2.1.1. Religious Affiliation

Respondents were asked if they had a religious affiliation, and if so, which the variable used for the analysis was dichotomous, with 1 = yes (including Catholic, Protestant, Evangelical, Jewish, Christian, or other) and 2 = no.

#### 2.1.2. Educational Level

Educational level was assessed with four possible responses: 1 = none or only elementary school (0–6 years), 2 = junior high school (7–9 years), 3 = high school (10–12 years), and 4 = undergraduate or graduate studies (13 or more years).

#### 2.1.3. Socioeconomic Index

An indicator was constructed using socioeconomic data, following the method of Gutiérrez et al. (2015), which considered ownership of goods (home, automobile, computer, DVD player, microwave oven) and access to services (internet, cable, and telephone). The index included five levels based on percentile distribution, classified into three groups: 1 = low and low-medium, 2 = medium and high-medium, and 3 = high [12].

#### 2.1.4. Symptoms of Mania/Hypomania

A positive response was defined as having been diagnosed with bipolar disorder or meeting the following two criteria: (1) having felt unusually happy, irritable, energetic, or hyperactive for three days or more, and (2) not having needed much sleep, without feeling tired, or having more energy than normal.

#### 2.1.5. Psychotic Symptoms 

A positive response was defined as having been diagnosed with schizophrenia or meeting the following two criteria: (1) having experienced a period of hearing voices when no one was present or having visions or seeing things that others could not see, and (2) having had ideas or beliefs that others did not share or that turned out not to be true [8,9].

#### 2.1.6. Anxiety Symptoms

A positive response was defined as having had the following three symptoms: (1) having experienced a sudden feeling of anxiety or fear; (2) having, as part of that experience, an accelerated heart rate, chest pains, shortness of breath, a choking sensation, nausea, sweating, weakness, or the fear of going crazy or dying; and (3) having these feelings worsen or intensify in the first ten minutes [8,9].

#### 2.1.7. Depression Symptoms

A positive response was defined as “having ever felt depressed, sad, or discouraged almost every day for two weeks or more” and also meeting one of the following criteria: (1) having ever lost most or all interest in normal activities for two weeks or more, (2) having had feelings of uselessness or guilt during that period, or spent a lot of time with thoughts of death, suicide, or self-harm, or (3) having noted significant changes in appetite during that period, or unexpected gain or loss in weight, changes in normal sleep patterns, or difficulty concentrating [8,9].

#### 2.1.8. Obsessive/Compulsive Symptoms

A positive response was defined as having had the following two symptoms: (1) having ever had repetitive thoughts or images, much more exaggerated than ordinary worries, that could not be stopped, and (2) having repeated certain behaviors over and over for an hour or more a day [8,9].

#### 2.1.9. Post-Traumatic Stress Symptoms

A positive response was defined as meeting the following two criteria: (1) having experienced a traumatic event that felt life-threatening, and (2) having experienced strong images or memories of traumatic events that return suddenly, such as uncontrollable thoughts or recurrent nightmares [8,9].

#### 2.1.10. Suicide Attempt

This variable was evaluated with the question: “In the last 12 months, have you tried to take your own life?” The variable was dichotomous, considering that there was a suicide attempt where respondents answered positively.

#### 2.1.11. Pathological Gambling

This variable was defined as recurrent and persistent dysfunctional gambling behavior that causes a clinically significant deterioration or illness, where an individual presents four or more symptoms in the previous 12 months, according to the criteria of the DSM-5 [11,13]. 

In addition, a variable was created with different groups of interest to this study, based on the presence of certain symptoms or comorbidity. These were:

#### 2.1.12. Comorbidity

The persons in this group met two criteria. The first was that they had at least one of the symptoms of mental illness: mania/hypomania, psychosis, anxiety, depression, obsession/compulsion, post-traumatic stress, suicide attempt, or pathological gambling. The second was that they had an alcohol, drug, or tobacco use disorder (SUD).

#### 2.1.13. Mental Illness Symptomatology

Participants in this group presented positive symptomatology for at least one of the previously mentioned mental health symptoms, but not SUD.

#### 2.1.14. Substance Use Disorder (SUD)

Participants in this group had none of the mental health symptoms described but did indicate having three or more symptoms of SUD during a single year at some point in their lives, based on the DSM-IV-TR [14], either with illegal or prescription drugs or with alcohol. Tobacco use disorder was evaluated using the scale and criteria of Fagerström [15].

#### 2.1.15. Drug Use without SUD

Participants in this group included those who had ever in their lives used a legal drug (alcohol or tobacco), an illegal drug, or an unprescribed medical drug, but without meeting the criteria for SUD and without presenting any mental illness symptomatology.

#### 2.1.16. Group without Mental Illness Symptomatology or Substance Use

This category included participants who said they had never used any legal drug (alcohol or tobacco), illegal drug (marijuana, cocaine, crack, inhalants, hallucinogens, or heroin), or unprescribed medical drug, and who had never presented any of the mental illness symptomatology described in this section.

### 2.2. Ethical Considerations

All the protocols for this study were approved by the Ethics Committees of the Instituto Nacional de Psiquiatría Ramón de la Fuente Muñiz (Approval No. CEI/C/083/2015) and the Instituto Nacional de Medicina Genómica (Approval No. 01/2017/I).

### 2.3. Statistical Analysis

Statistical analyses were carried out using the program STATA, version 13. Estimations were obtained of the prevalence of each symptomatology with an analysis of prevalence ratios (PR) based on generalized linear models (GLM) with log-link and binomial distribution. A multinomial logistic regression model was also carried out, where the dependent variable was that defining the substance use and comorbidity groups and the reference group that without symptomatology or use. These procedures allowed for better predictors and control of an important number of variables.

## 3. Results

### 3.1. Sample Characteristics

The majority of the participant population was aged 18–29 (30.8%) or 40–65 (34.9%), and 91% reported a religious affiliation. Almost half had a low or medium-low socioeconomic index (48.5%), and only 12.7% had an undergraduate education or more.

### 3.2. Mental Illness Symptomatology

As shown in Table 1, the most common symptoms reported by respondents are mania/hypomania (7.9%), depression (6.4%), and post-traumatic stress (5.7%). A total of 56.7% report having used a drug without SUD, 3.9% have experienced an SUD (5.4% alcohol, 0.8% tobacco, and 1.3% medical or illegal drugs), 15.9% report symptoms of mental illness, and 2.9% comorbidity of SUD and symptoms of mental illness. Women exceed men in the prevalence of depression (7.7% vs. 5.0%), anxiety (4.7% vs. 3.0%), and attempted suicide (1.5% vs. 0.6%); men report a greater frequency of pathological gambling (0.7% vs. 0.1%) and SUD (7.1% vs. 0.9%), with a slightly higher rate of psychotic symptoms (2.2% vs. 1.7%). There are no significant differences in the frequencies of mania/hypomania (7.7% in men vs. 8.0% in women), obsessive–compulsive disorder (2.3% vs. 2.2%), or post-traumatic stress (5.6% vs. 5.8%).

TUD is associated with mania/hypomania (19.2% with TUD vs. 7.8% without), anxiety (8.5% vs. 3.8%), depression (3.9% vs. 2.2%), and post-traumatic stress (16.6% vs. 5.6%). AUD or DUD are significantly associated with all pathologies analyzed, and AUD is also associated with suicide attempt. Those with TUD present more disorders (38.2% reported one or more) than those without TUD (17.8%); the figures for AUD are 40.2% and 16.7%, respectively, and for DUD 69.5% and 17.3%, respectively (Table 2).

### 3.3. Factors Associated with Mental Illness Symptomatology

A multiple regression model of prevalence ratios (PR) was carried out using various sociodemographic variables as predictors and different types of mental illness symptomatology as criterion variables (Table 3. The results show a lesser risk of presenting mania/hypomania symptomatology for those aged 40 and over (6.0%; PR = 0.71, 95% CI [0.53, 0.95]) and those with an undergraduate education or more (5.0%; PR = 0.60, 95% CI [0.40, 0.91]) than for those aged 12–17 (9.3%) or those with an elementary school education or less (7.4%). There is a lesser risk of psychotic symptoms for those aged 40 and over (1.5%; PR = 0.56, 95% CI [0.34–0.95]), a high school education (1.4%; PR = 0.55, 95% CI [0.32, 0.97]), or medium socioeconomic level (1.3%; PR = 0.56, 95% CI [0.35, 0.89]) than for those aged 12–17 (2.8%), those with an elementary school education or less (2.3%), or those with a low or medium-low socioeconomic level (2.3%).

Sex is the only predictive variable for symptoms of anxiety and depression. Men have a lower risk of presenting this symptomatology (anxiety 3.0%, PR = 0.63, 95% CI [0.47, 0.84]; depression 5.0%, PR = 0.63, 95% CI [0.50, 0.80]) than women (4.7% and 7.7%). Participants who report having no religion have a higher risk of symptoms of obsessive–compulsive disorder (4.4%, PR = 2.04, 95% CI [1.20, 3.48]), while those with a medium socioeconomic level have a lower risk (1.5%, PR = 0.60, 95% CI [0.38, 0.94]) than those with a low or medium-low level (2.6%). Young people aged 18–29 have a higher risk of presenting post-traumatic stress disorder (6.8%, PR = 1.54, 95% CI [1.10, 2.17]) than those aged 12–17 (4.5%).

Finally, there are significant differences by sex and educational level for suicide attempt and pathological gambling (Table 3b). For both indicators, those with an undergraduate education or more have a lower risk of presenting these problems (suicide attempt: 0.1%, PR = 0.13, 95% CI [0.03, 0.55]; pathological gambling: 0.0%, PR = 0.08, 95% CI [0.01, 0.51]) than those aged 12–17 (1.4% and 0.8%, respectively). Men have a lower risk for suicide attempt (0.6%, PR = 0.39, 95% CI [0.22, 0.70]) than women (1.5%), and a risk up to 10.49 times higher for pathological gambling (0.7%, PR = 11.49, 95% CI [3.40, 38.88]) than women (0.1%).

### 3.4. Factors Associated with Comorbidity

A multinomial logistic regression model was carried out to analyze the association of various factors with drug use and comorbidity, using the population that reported no drug use or mental illness symptomatology as the base category (Table 4). With respect to the base category, men have a risk 1.65 times greater than women for using drugs without symptoms of SUD, 18.9 times greater for presenting SUD, a 59% greater risk of presenting any symptom of mental illness, and a risk 10.8 times greater of having a comorbidity. Participants over 18 have a greater risk of presenting the four conditions analyzed than those who are younger, with the highest risk for SUD: 34.27 times greater in those aged 18–29, 34.75 times greater in those aged 30–39, and 54.36 times greater in those aged 40–65. Those reporting no religion have a 59% greater risk of presenting a symptom of mental illness. Those with junior high school and undergraduate education or more have a risk that is 39% and 72% greater, respectively, of presenting SUD, while for those with high school, the risk is 2.06 times greater than for those with elementary or no education, and their risk for SUD and symptomatology of mental illness is 1.44 and 1.43 times higher, respectively. Those with an undergraduate education or more have a greater risk of comorbidity than those with elementary or no education. Finally, those with a medium-high or high socioeconomic level have a 50% and 49% greater risk, respectively, of presenting without SUD and mental illness symptomatology.

## 4. Discussion

Psychiatric or mental health disorders have increased in recent years. Studies have been carried out in Mexico that indicate their prevalence in different populations, such as the general population, adolescents, and university students [2,3,16], but there is little current data. This study offers a view of the problem in urban and rural areas of the country in the population aged 12–65 prior to the COVID-19 pandemic.

Before proceeding to the discussion of the findings, the limitations of this study should be recognized. The results come from screening scales and not from diagnostic instruments; however, the scale been used has shown its consistency in estimating the frequency of mental illness symptoms in the general population and its utility in identifying those symptoms during the lifetime of those who respond [8,9,17]. The Diagnostic Interview for Psychosis and Affective Disorders (DI-PAD) aids mental health symptomatology, and it has been shown to have a good correlation coefficient with psychiatric diagnoses in large samples of psychiatric genetics (9). It is also important to note that the data were obtained from a self-assessment by the persons interviewed, and it is to be expected that a proportion of these persons present symptomatology but have not been diagnosed.

The Survey of Psychiatric Epidemiology (Encuesta de Epidemiología Psiquiátrica, ENEP) showed that 83% of persons with mental health problems sought help once or twice in their lives. However, only 22% received minimally appropriate care for persons with a diagnosis, which includes at least four visits per year to any type of provider, or at least two visits and any type of medication, or to be in treatment at the moment of the interview. This low figure is due mainly to the lack of access to mental health services. The ENEP found an average of 13 years between diagnosis and treatment of major depression, and 30 years for anxiety disorders [18], making clear the limitations of treatment center records and the need for data from national surveys. 

The information compiled in this study is highly useful for guiding public policy. The results find that 15.9% of respondents report symptoms of a mental health disorder at some time in their lives. The three most common symptoms are mania/hypomania (7.9%), followed by depression (6.4%), and post-traumatic stress (5.7%). The ENEP found a similar prevalence for depression (7.2%), but a lower prevalence for post-traumatic stress, which is to be expected, given that it was estimated before the increase in homicides and other forms of violence that are associated with this disorder [19]. Persons with SUD show greater comorbidity with mental illness, which is also consistent with what has been reported [6]. Compared to what has been reported in Latin America using the CIDI, our estimates for depression are lower as compared to Sao Paulo Brazil (10%) and higher than those reported in Argentina (3.7%), Colombia (5.3% and Peru (2.7%). Though similar to the rates reported for Mexico in 2003 also using the CIDI (3.7%) [2]. Which is, as mentioned before, expected due the changes in risk factors related to violence and increased poverty in Mexico, since that study [20].

The analysis of risk factors found that women with symptomatology of mental illness more commonly reported having a religion, a lower educational level, and a lower socioeconomic level, as has been reported in other studies [21,22]. Other studies have likewise found an association between religious belief and both unipolar depressive disorders and generalized anxiety [23].

Also consistent with prior studies carried out in Mexico is the finding that men show greater prevalence of substance use and a greater comorbidity between mental disorders and both alcohol and other substance use and pathological gambling. Women report a greater frequency of suicide attempt, a greater prevalence of mental illness symptomatology, and more depression and anxiety than men [2,5,10]. This is the first time the epidemiological frequency of obsessive–compulsive disorder has been analyzed on the national level, and this frequency coincides, as expected, with that described in other populations [17].

Telephone and online surveys carried out during the COVID-19 pandemic lockdown found a growth in the symptomatology of mental illness [24,25]. Though measured with different scales, comparisons with information available after COVID suggest increased anxiety and depression rates. The National Survey on Health and Nutrition 2021 [26] revealed that 10% of the population interviewed reported anxiety and 7.5% depression due to COVID, rates higher than our estimations (3.9% and 6.4%, respectively) before COVID. This increase is expected due to the known impact of COVID on Mental Health. Telephone Interviews reported higher prevalences of these two disorders, reaching a third of the population [25,27]. Data from this study, combined with information obtained during the pandemic, point to the importance of attending to an important problem affecting an important part of the population [28].

## 5. Conclusions

The results find that 15.9% of respondents reported symptoms of a mental health disorder at some time in their live. The three most common symptoms were of mania/hypomania (7.9%), followed by depression (6.4%), and post-traumatic stress (5.7%). In addition, people with a high school education had higher risk factors for DUD, SUD, and mental health symptoms comorbid with psychiatric symptoms. Results that despite coming from screening scales, clearly inform us of the mental health of Mexicans.

## 6. Limitations

A limitation of the study that we must point out is that the evaluation of psychiatric symptoms was obtained with the DI-PAD screening instrument, which only allows us to know the symptoms and not the diagnosis. So the definition of the comorbidity group is only an approximation and not the exact meaning of it, which is indicated in the Section 2.

## Figures and Tables

**Table 1:** Sociodemographic characteristics of the Mexican population aged 12–65.

	Men			Women				Total		
	n	N (Population)	%	n	N (Population)	%	*p*	n	N (Population)	%
Age (years)										
12–17	1592	7,266,945	17.8	1485	7,059,359	15.9	<0.001	3077	14,326,304	16.8
18–29	1158	12,857,659	31.5	1772	13,437,665	30.3		2930	26,295,324	30.8
30–39	815	6,246,013	15.3	1564	8,667,240	19.5		2379	14,913,253	17.5
40–65	1917	14,509,534	35.5	2827	15,217,642	34.3		4744	29,727,177	34.9
Religious affiliation										
Any religion	4808	36,196,886	88.5	7125	41,363,126	93.2	<0.001	11,933	77,560,013	91
None	674	4,683,265	11.5	523	3,018,780	6.8		1197	7,702,045	9
Educational level										
None/elementary	1455	8,845,522	22.2	2103	10,788,478	24.9	<0.001	3558	19,634,000	23.6
Junior high school	2223	14,460,140	36.3	3262	17,245,128	39.9		5485	31,705,268	38.1
High school	1149	10,702,516	26.8	1469	10,528,997	24.3		2618	21,231,513	25.5
Undergraduate or more	508	5,877,411	14.7	555	4,685,774	10.8		1063	10,563,184	12.7
Socioeconomic index										
Low and low-medium	2958	19,010,487	46.6	4347	22,279,018	50.2	<0.001	7305	41,289,505	48.5
Medium	1234	9,648,461	23.6	1629	9,484,551	21.4		2863	19,133,012	22.5
Medium-high and high	1280	12,153,136	29.8	1663	12,591,119	28.4		2943	24,744,255	29.1
Substance use disorder										
Tobacco	54	579,073	1.4	24	123,440	0.3	<0.001	78	702,513	0.8
Alcohol	466	4,004,146	9.8	77	596,554	1.3	<0.001	543	4,600,700	5.4
Drugs	125	918,174	2.2	30	189,401	0.4	<0.001	155	1,107,576	1.3
Mental illness symptomatology										
Mania/hypomania	438	3,165,625	7.7	611	3,541,184	8.0	0.612	1049	6,706,809	7.9
Psychotic symptoms	141	893,715	2.2	157	737,605	1.7	0.029	298	1,631,320	1.9
Anxiety	204	1,220,036	3.0	402	2,094,576	4.7	<0.001 *	606	3,314,612	3.9
Depression	274	2,025,149	5.0	579	3,424,118	7.7	<0.001	853	5,449,268	6.4
Obsessive-compulsive	148	943,633	2.3	202	957,956	2.2	0.593	350	1,901,589	2.2
Post-traumatic stress	281	2,271,000	5.6	456	2,564,727	5.8	0.586	737	4,835,727	5.7
Suicide attempt	41	242,576	0.6	121	672,639	1.5	<0.001	162	915,214	1.1
Pathological gambling	35	305,201	0.7	8	29,856	0.1	<0.001	43	335,057	0.4
SUD and mental illness symptomatology										
Neither mental illness symptomatology nor drug use	987	5,613,132	13.7	2305	11,946,913	26.9	<0.001	3292	17,560,045	20.6
Drug use without SUD ^1^	3183	24,923,360	61.0	3748	23,440,548	52.8		6931	48,363,908	56.7
SUD ^1^	331	2,911,150	7.1	54	379,802	0.9		385	3,290,952	3.9
Mental illness symptomatology	742	5,418,548	13.3	1476	8,155,460	18.4		2218	13,574,008	15.9
SUD and mental illness symptomatology	239	2,013,960	4.9	65	459,185	1.0		304	2,473,145	2.9

^1^ Including alcohol, tobacco, unprescribed medical drugs, and any illegal drug. N = 84,357,412 (men 40,336,732, women 44,020,681); n = 13,130 (men 6297, women 6835). * *p*-value of X^2.^

**Table 2:** Presence of mental illness symptomatology and SUD on tobacco, alcohol, and drugs.

	TUD					AUD (Ever)					DUD (Ever)				
	With TUD		Without TUD			With AUD		Without AUD			With DUD		Without DUD		
	n	%	n	%	*p* *	n	%	n	%	*p* *	n	%	n	%	*p* *
Mania/hypomania	21	19.2	1012	7.8	<0.001	149	21	884	7.1	<0.001	61	35.6	972	7.5	<0.001
Psychotic symptoms	3	2.8	248	1.9	0.509	44	6.2	207	1.7	<0.001	37	21.6	214	1.7	<0.001
Anxiety	9	8.5	501	3.8	0.016	76	10.7	434	3.5	<0.001	33	19.2	478	3.7	<0.001
Depression	18	16.4	821	6.3	<0.001	110	15.5	729	5.9	<0.001	41	24.1	798	6.2	<0.001
Obsessive-compulsive	4	3.9	289	2.2	0.298	55	7.7	238	1.9	<0.001	28	16.1	265	2	<0.001
Post-traumatic stress	18	16.6	727	5.6	<0.001	75	10.5	670	5.4	<0.001	31	18.4	713	5.5	<0.001
Suicide attempt	2	1.5	139	1.1	0.439	14	1.9	127	1	0.017	13	7.9	127	1	<0.001
Pathological gambling	1	0.9	51	0.4	0.379	10	1.3	42	0.3	<0.001	2	1.3	49	0.4	0.096

TUD: Tobacco Use Dependence; AUD: Alcohol Use Dependence; DUD: Drug Use Dependence. * *p*-value of X^2^.

**Table 3:** Predictors of mental illness symptomatology.

	Mania/Hypomania					Psychotic Symptoms					Anxiety					Depression				
	n	% ^1^	PR	95% CI	*p*	n	% ^1^	PR	95% CI	*p*	n	% ^1^	PR	95% CI	*p*	n	% ^1^	PR	95% CI	*p*
Sex																				
Female	545	8.0	1.0			114	1.7	1.0			323	4.7	1.0			527	7.7	1.0		
Male	487	7.7	0.97	0.78–1.21	0.807	138	2.2	1.32	0.91–1.92	0.142	188	3.0	0.63	0.47–0.84	0.001	312	5.0	0.63	0.50–0.80	<0.001
Age (years)																				
12–17	205	9.3	1.0			61	2.8	1.0			71	3.2	1.0			138	6.3	1.0		
18–29	367	9.1	1.03	0.78–1.35	0.835	76	1.9	0.74	0.45–1.19	0.215	158	3.9	1.19	0.79–1.77	0.404	283	7.0	1.16	0.81–1.66	0.410
30–39	186	8.1	0.94	0.66–1.34	0.735	44	1.9	0.71	0.42–1.20	0.203	87	3.8	1.12	0.75–1.66	0.573	115	5.0	0.81	0.56–1.17	0.258
40–65	274	6.0	0.71	0.53–0.95	0.020	70	1.5	0.56	0.34–0.95	0.030	194	4.2	1.30	0.90–1.88	0.157	302	6.6	1.03	0.73–1.44	0.876
Religious affiliation																				
Any religion	904	7.6	1.0			215	1.8	1.0			460	3.9	1.0			741	6.2	1.0		
None	129	10.8	1.33	0.99–1.79	0.054	36	3.1	1.56	0.92–2.67	0.102	50	4.2	1.15	0.77–1.72	0.496	98	8.2	1.40	0.99–1.97	0.054
Educational level																				
None/elementary	222	7.4	1.0			70	2.3	1.0			142	4.7	1.0			213	7.1	1.0		
Junior high school	401	8.2	1.02	0.76–1.36	0.897	104	2.1	0.85	0.55–1.33	0.476	177	3.6	0.83	0.58–1.19	0.321	321	6.6	0.92	0.69–1.24	0.590
High school	317	9.7	1.15	0.83–1.61	0.405	45	1.4	0.55	0.32–0.97	0.040	121	3.7	0.88	0.58–1.33	0.541	183	5.6	0.77	0.54–1.08	0.132
Undergraduate or more	81	5.0	0.60	0.40–0.91	0.016	30	1.9	0.88	0.45–1.70	0.696	62	3.8	0.95	0.57–1.58	0.841	92	5.7	0.80	0.51–1.24	0.317
Socioeconomic index																				
Low and low-medium	519	8.2	1.0			149	2.3	1.0			261	4.1	1.0			403	6.3	1.0		
Medium	223	7.6	0.94	0.69–1.27	0.677	39	1.3	0.56	0.35–0.89	0.015	121	4.1	0.98	0.70–1.38	0.922	215	7.3	1.20	0.91–1.59	0.200
Medium-high and high	289	7.6	0.99	0.78–1.27	0.949	61	1.6	0.74	0.49–1.12	0.150	128	3.4	0.81	0.60–1.09	0.171	217	5.7	0.95	0.71–1.27	0.719
	Obsessive-Compulsive					Post-traumatic Stress					Suicide Attempt					Pathological Gambling				
	n	% ^1^	PR	95% CI	*p*	n	% ^1^	PR	95% CI	*p*	n	% ^1^	PR	95% CI	*p*	n	% ^1^	PR	95% CI	*p*
Sex																				
Female	148	2.2	1.0			395	5.8	1.0			104	1.5	1.0			5	0.1	1.0		
Male	145	2.3	1.05	0.74–1.49	0.776	350	5.6	0.96	0.76–1.21	0.727	37	0.6	0.39	0.22–0.70	0.001	47	0.7	11.49	3.40–38.88	< 0.001
Age (years)																				
12–17	55	2.5	1.0			100	4.5	1.0			32	1.4	1.0			10	0.5	1.0		
18–29	117	2.9	1.23	0.80–1.89	0.344	275	6.8	1.54	1.10–2.17	0.013	41	1.0	0.85	0.43–1.70	0.654	14	0.3	1.09	0.37–3.16	0.881
30–39	39	1.7	0.74	0.42–1.31	0.304	119	5.2	1.22	0.85–1.76	0.278	23	1.0	0.75	0.36–1.56	0.436	5	0.2	0.56	0.12–2.54	0.455
40–65	82	1.8	0.80	0.53–1.21	0.290	251	5.5	1.33	0.91–1.95	0.143	46	1.0	0.70	0.37–1.31	0.267	22	0.5	1.15	0.26–5.08	0.853
Religious affiliation																				
Any religion	240	2.0	1.0			667	5.6	1.0			126	1.1	1.0			46	0.4	1.0		
None	53	4.4	2.04	1.20–3.48	0.009	78	6.6	1.16	0.79–1.71	0.455	15	1.3	1.37	0.55–3.44	0.502	6	0.5	1.03	0.40–2.64	0.946
Educational level																				
None/elementary	67	2.2	1.0			153	5.1	1.0			42	1.4	1.0			23	0.8	1.0		
Junior high school	119	2.4	1.02	0.68–1.54	0.926	289	5.9	1.17	0.82–1.66	0.394	60	1.2	0.88	0.51–1.53	0.650	14	0.3	0.40	0.10–1.56	0.187
High school	69	2.1	0.82	0.50–1.34	0.425	215	6.6	1.20	0.84–1.70	0.312	32	1.0	0.76	0.37–1.56	0.448	13	0.4	0.56	0.14–2.25	0.413
Undergraduate or more	32	2.0	0.77	0.38–1.57	0.475	81	5.0	0.84	0.51–1.36	0.466	2	0.1	0.13	0.03–0.55	0.006	1	0.0	0.08	0.01–0.51	0.008
Socioeconomic index																				
Low and low-medium	163	2.6	1.0			339	5.3	1.0			86	1.3	1.0			39	0.6	1.0		
Medium	44	1.5	0.60	0.38–0.94	0.027	177	6.0	1.09	0.82–1.45	0.549	23	0.8	0.63	0.35–1.14	0.124	6	0.2	0.32	0.08–1.23	0.096
Medium-high and high	86	2.2	0.92	0.60–1.41	0.698	227	6.0	1.11	0.80–1.53	0.540	31	0.8	0.80	0.39–1.63	0.541	7	0.2	0.37	0.11–1.20	0.097

^1^ symptom % of each category.

**Table 4:** Multinomial logistic regression model of SUD and mental illness symptomatology.

	Drug Use without SUD ^1^					SUD ^1^					Mental Illness Symptomatology					SUD and Mental Illness Symptomatology (Comorbidity)				
Ref “neither symptomatology nor use”	n	%	OR	95% CI	*p*	n	%	OR	95% CI	*p*	n	%	OR	95% CI	*p*	n	%	OR	95% CI	*p*
Sex																				
Female	3610	52.8	1.0			58	0.9	1.0			1256	18.4	1.0			71	1.0	1.0		
Male	3838	61.0	2.65	2.21–3.17	<0.001	448	7.1	19.95	11.41–34.88	<0.001	834	13.3	1.59	1.28–1.98	<0.001	310	4.9	11.79	7.29–19.09	<0.001
Age (years)																				
12–17	664	30.1	1.0			13	0.6	1.0			402	18.2	1.0			20	0.9	1.0		
18–29	2527	62.4	7.81	6.06–10.06	<0.001	156	3.8	35.27	16.78–74.14	<0.001	674	16.6	3.45	2.66–4.49	<0.001	155	3.8	23.50	8.94–61.73	<0.001
30–39	1471	64.1	9.31	7.09–12.23	<0.001	77	3.4	35.75	13.76–92.90	<0.001	322	14.0	3.16	2.27–4.39	<0.001	83	3.6	23.68	9.06–61.88	<0.001
40–65	2786	60.9	8.96	7.18–11.17	<0.001	261	5.7	55.36	24.14–126.93	<0.001	693	15.1	3.42	2.58–4.54	<0.001	123	2.7	13.62	5.27–35.20	<0.001
Religious affiliation																				
Any religion	6756	56.6	1.0			459	3.8	1.0			1879	15.7	1.0			322	2.7	1.0		
None	692	58.4	1.42	0.97–2.08	0.069	48	4.0	1.39	0.75–2.59	0.300	211	17.8	1.59	1.08–2.34	0.019	59	5.0	2.02	0.97–4.22	0.061
Educational level																				
None/elementary	1524	50.4	1.0			128	4.2	1.0			467	15.4	1.0			124	4.1			
Junior high school	2509	51.4	1.39	1.14–1.70	0.001	197	4.0	1.60	0.79–3.22	0.192	796	16.3	1.29	1.00–1.66	0.055	128	2.6	0.82	0.47–1.41	0.465
High school	2099	64.2	3.06	2.33–4.00	<0.001	121	3.7	2.44	1.23–4.85	0.011	559	17.1	2.43	1.81–3.27	<0.001	91	2.8	1.32	0.64–2.73	0.456
Undergraduate or more	1137	69.9	1.72	1.13–2.61	0.011	44	2.7	0.85	0.38–1.88	0.684	230	14.2	1.24	0.77–1.99	0.382	22	1.4	0.33	0.14–0.74	0.007
Socioeconomic index																				
Low and low-medium	3338	52.5	1.0			261	4.1	1.0			974	15.3	1.0			226	3.6	1.0		
Medium	1698	57.6	1.24	0.98–1.56	0.070	123	4.2	1.20	0.70–2.05	0.518	484	16.4	1.25	0.99–1.59	0.064	74	2.5	0.82	0.48–1.40	0.465
Medium-high and high	2404	63.1	1.50	1.21–1.88	<0.001	122	3.2	1.08	0.59–1.96	0.802	627	16.5	1.49	1.16–1.93	0.002	81	2.1	0.93	0.53–1.62	0.795

^1^ symptom % of each category.
